# Evaluation of hearing and cochlear function by audiometric testing in patients with hyperemesis gravidarum

**DOI:** 10.11604/pamj.2015.20.231.5053

**Published:** 2015-03-12

**Authors:** Ahmet Kale, Arzu Yavuz, Adin Selçuk, Ömer Demirtas, Hasan Terzi, Selahattin Genç

**Affiliations:** 1 Kocaeli Derince Education and Research Hospital, Department of Obstetrics and Gynecology, Kocaeli, Turkey; 2 Kocaeli Derince Education and Research Hospital ENT Clinic, Kocaeli, Turkey; 3 Pamukkale University Faculty of Medicine Hospital, Department of Obstetrics and Gynecology, Denizli, Turkey

**Keywords:** Hyperemesis gravidarum, pregnancy, hearing loss, cochlear function

## Abstract

**Introduction:**

The aim of this study was to investigate cochlear functions in patients with hyperemesis gravidarum (HG).

**Methods:**

Twenty-nine HG patients (58 ears) and 31 healthy control subjects (62 ears) were included. Audiometry testings at 250 and 500 Hz and 1, 2, 4, 8, 10, 12, 14, 16 kHz were performed to the patients and controls.

**Results:**

Mean age of patients with HG was 26,5 ± 4,4 years and the mean age of control group was 28,0 ± 4,2 years. At the time of the tests mean gestational age of the HG group and controls were 9 and 11 weeks respectively. No differences were observed between the groups in tympanic membrane status, orother otolaringological evaluations. No significant differences were observed in audiometric tests at any frequencies between the groups (p values for all > 0.05).

**Conclusion:**

There was not a difference between pregnant cases with HG and cases with normal pregnancy in terms of audimetric tests. Cochlear functions are not affectedremarkably in women with HG.

## Introduction

Hyperemesis gravidarum(HG) is a severe, intractable complication of pregnancy. Hyperemesis is considered a rare complication of pregnancy with nausea, vomiting and dehidratation that affects approximately 0.3–2% of pregnancies [1]. HG can be associated with ketosis with weight loss > 5%, electrolyte disturbances and dehydration.

HG is the most common reason for hospitalization in first trimester and second most common cause for hospitalization in pregnancy [2, 3]. Adaptation to pregnancy necessitates various physiological changes in almost all organ systems. Intracellular and extracellular fluid changes, osmolality changes, and alterations in the immune system are among the crucial changes exhibited during pregnancy. These changes in pregnancy may have impacts on cochlear microcirculation and fluid balance in the cochlea; therefore, they may also alter hearing levels [4–6]. Audiology studies in pregnancy have shown a cochlear influence [6, 7], and this influence is associated with sensorineural hearing loss in low frequencies and tolerance problems. However, hearing tests normalize after birth. Postpartum normalization shows that the cochlear influence may be a physiological adaptation, or may be affected by changes during pregnancy. Although the etiology of hyperemesis gravidarum is still uncertain, suggested theories are similar to the etiology of cochlear damage. Herein, we aimed to determine if cochlear functions are affected in cases of HG.

## Methods

This study was designed as cross case-control study and performed between September of 2012 and November of 2013, in the Otolaryngology and Obstetrics-Gynecology Departments. All patients were informed and consents were obtained. The study was approved by our Institutional Research Ethical Committee. 31 healthy pregnant women were included as control group and 29 pregnancies with HG. The presence of ketonuria and intractable vomiting were defined as HG. The hearing tests and urine tests were performed on the same days. All HG patients and control group were monitored by the same investigator. All audiometric evaluations were assessed by the same audiometric, as well. Patients with systemic illnesses, maternal age >40 years, a history of ototoxic drug consumption, multiple pregnancies, history of ear disease or temporal bone fracture, history of cranial trauma, history of ear surgery, noise exposure, perforated tympanic membrane, Meniere's disease and metabolic diseases were excluded from the study. Otoscopic examinations were performed for all patients. Conventional pure tone and high frequency audiometry was done using an Interacoustics AC 40 audiometer and TDH 39P earphones (thresholds in dB NA) in a sound-treated cabin (Interacoustics Company, Denmark).

Bone and airway conduction thresholds were measured at frequencies of 250 and 500 Hz and 1, 2, 4, 8, 10, 12, 14, and 16 kHz. The speech recognition threshold was measured, followed by the percentage rate of speech recognition, in an acoustic booth in order to avoid interference from extraneous noise. The exclution criteria after audometric evaluation were one of the following: (a) evidence of a perforated tympanic membrane, (b) evidence of middle ear pathology, (c) presence of a flat tympanogram, (d) absence of acoustic reflexes at 1 kHz with contralateral stimulation, or (e) an air-bone gap of 5 dB at any frequency. Thus, one patient was excluded from the study, due to a history of trauma.

Normal middle ear function was defined by the immittance and acoustic reflex results using an InteracousticsAZ 26 Clinical impedance meter. The patients and controls who had normal peak compliance, peak pressure, gradient, ear canal volume, and acoustic reflexes obtained by immittance measurements, as defined by the American Speech Language and Hearing Association, were included in the study [8, 9]. All analyses were carried out using PASW software, (SPSS version 18, Chicago, Illinois, USA). Normal distribution and homogeneity of variance tests were performed. For the overall group comparisons (patients with HG and the controls) the Mann-Whitney U test for non -parametric values and student's t -test for parametric values were used for statistical comparisons. Pearson correlation test was used to evaluate the relationships between the quantitative parameters. P value of < 0.05 was considered to be significant.

## Results

**Figure 1:**
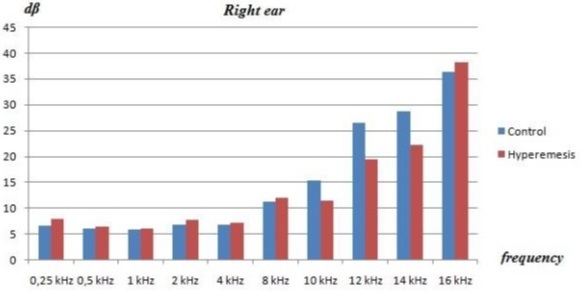
Pure tone thresholds of the patient and controls (right ear)

**Figure 2:**
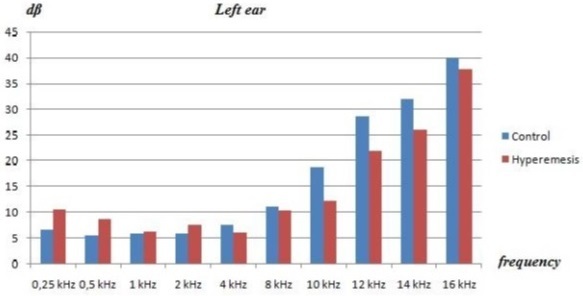
Pure tone thresholds of the patient and controls (left ear)

The mean age of the patients with HG was 26.5 ± 4.4 years, and the mean age of the control group was 28.0 ± 4 .2 years. At the time of the tests, the mean gestational ages of the HG group and controls were 9 and 11 weeks, respectively. Otoscopic examinations were normal in all patients, and no statistical differences were observed between HG patients and healthy pregnant controls in the baseline characteristics, such as age, duration of pregnancy, or obstetric history. There was no statistical differences between the groups in tympanic membrane status or other otolaryngological evaluations. Normal peak compliance, peak pressure, gradient, ear canal volume, and acoustic reflexes were obtained by immittance measures in the patients and controls. The average pure tone bone conduction hearing thresholds in both groups at each frequency are shown at Table 1. No significant differences were observed in the audiometric tests at any frequencies between the groups (p values for all > 0.05). Because there were no air-bone gaps in the participants, only the bone conduction thresholds were taken into consideration. There were no significant differences in terms of the pure tone thresholds of the patients and controls (p > 0.05) Figure 1 and Figure 2. Sensorineural hearing loss was found in 10 patients (35%), and it was bilateral in seven and unilateral in three patients.

**Table 1:** Audiological findings (Average pure tone bone conduction hearing thresholds) in hyperemesis gravidarum group and healthy pregnant group at each frequency

Frequency (kHz)	Control (n:31)		Hyperemesis gravidarum (n:29)		
Right Ear	Mean-Std	Min-Max	Mean-Std	Min-Max	*p values*
**0,25**	6,6±2,9	5-15	7,93±5,431	5-30	*0,398*
**0,5**	6,1±2,4	5-15	6,55±2,707	5-15	*0,469*
**1**	5,8±1,8	5-10	6,03±2,061	5-10	*0,651*
**2**	6,9±6,6	5-40	7,76±5,276	5-25	*0,178*
**4**	6,9±7,3	5-45	7,24±4,137	5-20	*0,171*
**8**	11,2±9,3	5-35	12,07±10,396	5-45	*0,732*
**10**	15,4±18,09	5-85	11,55±8,356	0-30	*0,810*
**12**	26,6±23,6	5-90	19,48±14,537	0-60	*0,496*
**14**	28,7±25,3	5-80	22,24±17,453	0-70	*0,643*
**16**	36,4±26,08	5-80	38,28±19,968	5-80	*0,749*
**Left Ear**	**Mean-Std**	**Min-Max**	**Mean-Std**	**Min-Max**	
**0,25**	**6,7±3,5**	**5-15**	**10,52±7,600**	**5-30**	***0,026***
**0,5**	**5,6±1,7**	**5-10**	**8,62±5,493**	**5-25**	***0,008***
**1**	5,9±3	5-20	6,38±2,638	5-15	*0,286*
**2**	5,9±3,7	5-25	7,59±5,766	5-25	*0,135*
**4**	7,4±7,7	5-45	6,21±2,883	5-15	*0,991*
**8**	11,2±11,3	5-65	10,34±7,062	5-25	*0,968*
**10**	18,8±19	5-75	12,24±7,019	0-30	*0,472*
**12**	28,7±24,8	5-90	21,90±16,280	0-60	*0,493*
**14**	31,9±25,4	5-80	26,03±20,804	0-70	*0,551*
**16**	40,1±27,3	5-80	37,93±23,6	5-80	*0,876*

## Discussion

Nausea and vomiting in pregnancy are reported in 70–85% of pregnant women [1]. Most patients with nausea and vomiting resolve spontaneously during first trimester but a small percentage of cases progresses to HG. HG is the most common reason for hospitalization in first trimester and second most common cause for hospitalization in pregnancy [2, 3]. The etiology and pathogenesis of HG remains unknown; however, the potential roles of pregnancy-related hormones, infections, and immunological, psychological, metabolic, and anatomical causes for HG have been analyzed in the literature [10]. These etiological factors have similarities with those of cochlear dysfunction, which is an important health issue in developing countries. So we aimed to determine if cochlear functions are affected in cases of HG. In our study, there were no significant differences observed in the audiometric tests at any frequencies between the groups.

According to the WHO, around 360 million people are at risk, and of these, 80% are from countries with low socio-economic levels [11]. Cochlear dysfunction is rare during pregnancy, and the incidence and prevalence have not yet been designated. Data on how pregnancy affects cochlear functions is limited. Some audiological studies have shown pregnancy to have an effect on cochlear functions, and that it is particularly associated with sensorineural hearing loss in low frequencies and tolerance problems [6, 7]. Hearing tests normalize after birth in cases with a normal course of pregnancy. In our study, we only compared the hearing levels between pregnant women and women with HG during pregnancy. A further study showing postpartum hearing levels may clarify this postpartum normalization, which suggests that the changes in cochlear functions may be a physiological adaptation. Physiological changes during pregnancy may influence the microcirculation or fluid balance of the cochlea, therefore, altering hearing levels [4–6]. Hou et al. presented cases with acute onset sensorineural hearing loss (SHL): one due to the dysregulation of cochlear fluid distribution secondary to the changes in the fluid balance and osmolality during pregnancy; the second due to an underlying disease which disturbed cochlear circulation [4]. Supporting the etiology suggested by Hou et al., Uchide et al. presented a case with Meniere disease, which had an increased frequency of attacks during pregnancy [8]. In light of these facts, cochlear changes may be secondary to the pregnancy related physiological adaptations of the inner ear [8, 9, 12]. Although the etiology of cochlear dysfunction remains elusive, vascular, viral, immunological, hematological, and hormonal factors are among the factors that are commonly held to be responsible [4, 13, 14]. Herein, we aimed to evaluate the audiometric results in cases with HG. In our study, there were no significant differences observed in the audiometric tests at any frequencies between the groups. The indifference may be because the changes in the immune system and fluid balance were involved in the etiopathogenesis of both conditions. In addition to the physiological changes of pregnancy, the immune system, hormonal, and metabolic status are also involved in HG. Hormonally and cellularly mediated changes in the immune system may occur during pregnancy, and physiological immune reactions to pregnancy may be associated with pregnancy related disorders. Immune mediated ovarian activation has a significant role in the etiology of HG [15]. Higher levels of fetal DNA, immunoglobulin G and M, C3, and C4 in the plasma of the cases with HG are among the evidence which supports the contribution of immunological mechanisms in HG [16–19]. Similarly, immunological factors come into prominence in cochlear pathologies.

The inner ear is easily affected by systemic diseases, and immunological disturbances are associated with many causes of auditory dysfunction [20, 21]. Various studies and case reports have shown the role of auto-antibodies against the labyrinth [22–25]. During pregnancy, cochlear changes first occur at low frequencies; however, the patients do not realize these changes. Our cases did not complain about hearing loss. Similarly, hearing loss at low frequencies is also the first finding in cases of pathological disturbances of the cochlea. There were no hearing differences between the groups at low frequencies in our study. If our study had compared the hearing values during and after pregnancy, we would be able to see the changes in hearing at low frequencies; however, in this study, we aimed to determine if cochlear functions are affected in cases with HG. Changes after vascular endothelial injury are irreversible, whereas physiological changes during pregnancy revert back to normal after giving birth. In conditions like preeclampsia, HELLP syndrome, and autoimmune disorders, where vascular damage is seen, permanent dysfunction occurs in the cochlea. This damage may be due to ischemia in the cochlear circulation. Ongoing hearing loss after birth shows that the ischemia is persistent. In cases with an uncomplicated course of pregnancy, cochlear changes revert back to normal since they occur due to alterations in the distribution of fluid and electrolytes.

## Conclusion

In conclusion, we found no differences between the pregnant cases with HG and cases with normal pregnancy in terms of audiometric testing. In addition to the physiological changes, different hormonal, immunological, and metabolic conditions are involved in HG. Although all of these factors cause changes with various levels of intensity, cochlear functions are not affected remarkably in women with HG.
